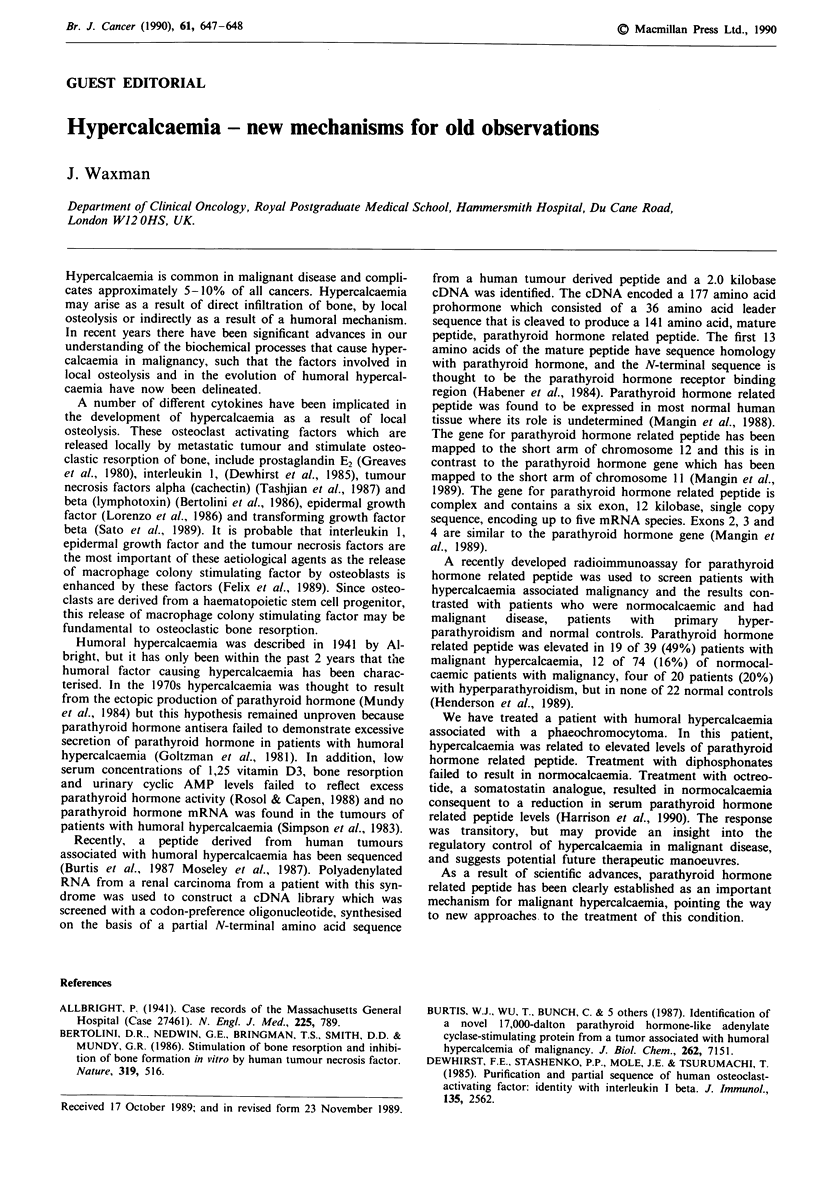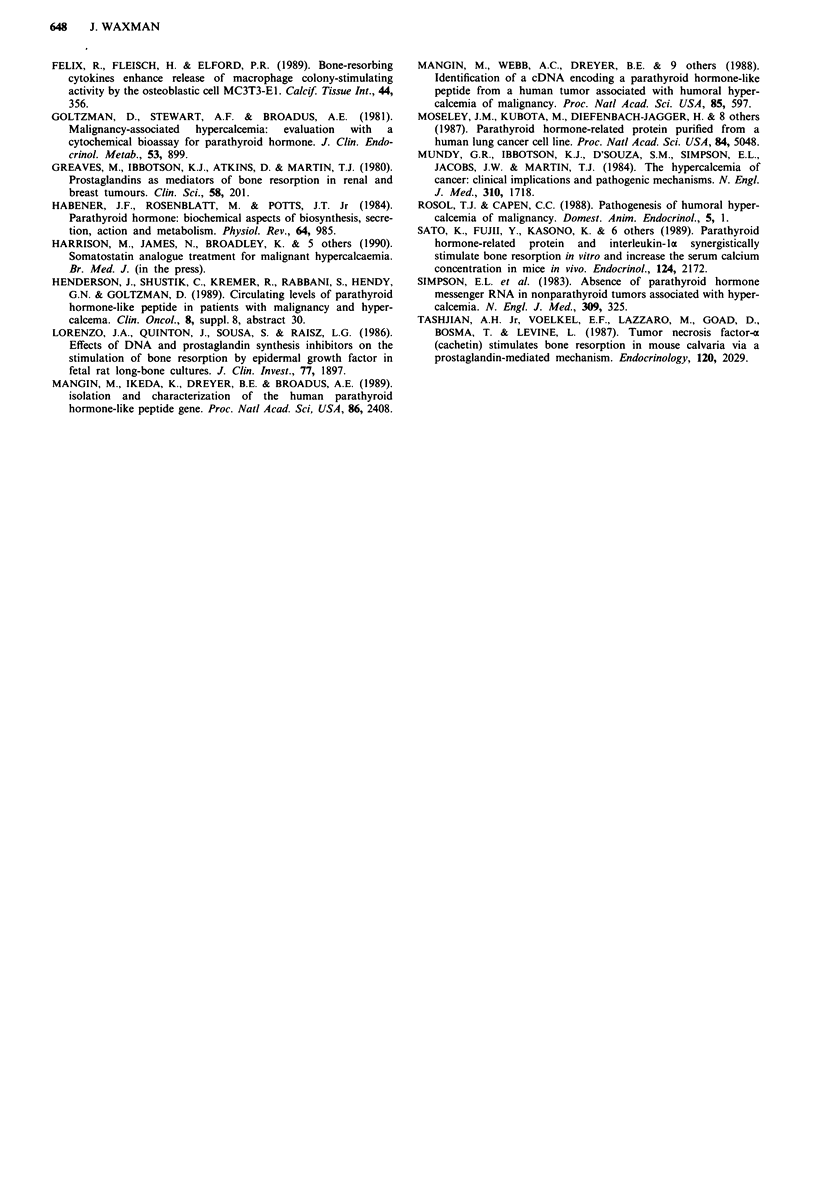# Hypercalcaemia--new mechanisms for old observations.

**DOI:** 10.1038/bjc.1990.145

**Published:** 1990-05

**Authors:** J. Waxman

**Affiliations:** Department of Clinical Oncology, Royal Postgraduate Medical School, Hammersmith Hospital, London, UK.


Br. J. Cancer (1990), 61, 647-648 © Macmillan Press Ltd., 1990

GUEST EDITORIAL

### Hypercalcaemia – new mechanisms for old observations

J. Waxman

*Department of Clinical Oncology, Royal Postgraduate Medical School, Hammersmith Hospital, Du Cane Road, London W12 0HS, UK.*

Hypercalcaemia is common in malignant disease and complicates approximately 5–10% of all cancers. Hypercalcaemia may arise as a result of direct infiltration of bone, by local osteolysis or indirectly as a result of a humoral mechanism. In recent years there have been significant advances in our understanding of the biochemical processes that cause hypercalcaemia in malignancy, such that the factors involved in local osteolysis and in the evolution of humoral hypercalcaemia have now been delineated.

A number of different cytokines have been implicated in the development of hypercalcaemia as a result of local osteolysis. These osteoclast activating factors which are released locally by metastatic tumour and stimulate osteoclastic resorption of bone, include prostaglandin $E_2$ (Greaves *et al.*, 1980), interleukin 1, (Dewhirst *et al.*, 1985), tumour necrosis factors alpha (cachectin) (Tashjian *et al.*, 1987) and beta (lymphotoxin) (Bertolini *et al.*, 1986), epidermal growth factor (Lorenzo *et al.*, 1986) and transforming growth factor beta (Sato *et al.*, 1989). It is probable that interleukin 1, epidermal growth factor and the tumour necrosis factors are the most important of these aetiological agents as the release of macrophage colony stimulating factor by osteoblasts is enhanced by these factors (Felix *et al.*, 1989). Since osteoclasts are derived from a haematopoietic stem cell progenitor, this release of macrophage colony stimulating factor may be fundamental to osteoclastic bone resorption.

Humoral hypercalcaemia was described in 1941 by Albright, but it has only been within the past 2 years that the humoral factor causing hypercalcaemia has been characterised. In the 1970s hypercalcaemia was thought to result from the ectopic production of parathyroid hormone (Mundy *et al.*, 1984) but this hypothesis remained unproven because parathyroid hormone antisera failed to demonstrate excessive secretion of parathyroid hormone in patients with humoral hypercalcaemia (Goltzman *et al.*, 1981). In addition, low serum concentrations of 1,25 vitamin D3, bone resorption and urinary cyclic AMP levels failed to reflect excess parathyroid hormone activity (Rosol & Capen, 1988) and no parathyroid hormone mRNA was found in the tumours of patients with humoral hypercalcaemia (Simpson *et al.*, 1983).

Recently, a peptide derived from human tumours associated with humoral hypercalcaemia has been sequenced (Burtis *et al.*, 1987 Moseley *et al.*, 1987). Polyadenylated RNA from a renal carcinoma from a patient with this syndrome was used to construct a cDNA library which was screened with a codon-preference oligonucleotide, synthesised on the basis of a partial *N*-terminal amino acid sequence from a human tumour derived peptide and a 2.0 kilobase cDNA was identified. The cDNA encoded a 177 amino acid prohormone which consisted of a 36 amino acid leader sequence that is cleaved to produce a 141 amino acid, mature peptide, parathyroid hormone related peptide. The first 13 amino acids of the mature peptide have sequence homology with parathyroid hormone, and the *N*-terminal sequence is thought to be the parathyroid hormone receptor binding region (Habener *et al.*, 1984). Parathyroid hormone related peptide was found to be expressed in most normal human tissue where its role is undetermined (Mangin *et al.*, 1988). The gene for parathyroid hormone related peptide has been mapped to the short arm of chromosome 12 and this is in contrast to the parathyroid hormone gene which has been mapped to the short arm of chromosome 11 (Mangin *et al.*, 1989). The gene for parathyroid hormone related peptide is complex and contains a six exon, 12 kilobase, single copy sequence, encoding up to five mRNA species. Exons 2, 3 and 4 are similar to the parathyroid hormone gene (Mangin *et al.*, 1989).

A recently developed radioimmunoassay for parathyroid hormone related peptide was used to screen patients with hypercalcaemia associated malignancy and the results contrasted with patients who were normocalcaemic and had malignant disease, patients with primary hyperparathyroidism and normal controls. Parathyroid hormone related peptide was elevated in 19 of 39 (49%) patients with malignant hypercalcaemia, 12 of 74 (16%) of normocalcaemic patients with malignancy, four of 20 patients (20%) with hyperparathyroidism, but in none of 22 normal controls (Henderson *et al.*, 1989).

We have treated a patient with humoral hypercalcaemia associated with a phaeochromocytoma. In this patient, hypercalcaemia was related to elevated levels of parathyroid hormone related peptide. Treatment with diphosphonates failed to result in normocalcaemia. Treatment with octreotide, a somatostatin analogue, resulted in normocalcaemia consequent to a reduction in serum parathyroid hormone related peptide levels (Harrison *et al.*, 1990). The response was transitory, but may provide an insight into the regulatory control of hypercalcaemia in malignant disease, and suggests potential future therapeutic manoeuvres.

As a result of scientific advances, parathyroid hormone related peptide has been clearly established as an important mechanism for malignant hypercalcaemia, pointing the way to new approaches to the treatment of this condition.

### References

ALLBRIGHT, P. (1941). Case records of the Massachusetts General Hospital (Case 27461). *N. Engl. J. Med.*, **225**, 789.

BERTOLINI, D.R., NEDWIN, G.E., BRINGMAN, T.S., SMITH, D.D. & MUNDY, G.R. (1986). Stimulation of bone resorption and inhibition of bone formation *in vitro* by human tumour necrosis factor. *Nature*, **319**, 516.

Received 17 October 1989; and in revised form 23 November 1989.

BURTIS, W.J., WU, T., BUNCH, C. & 5 others (1987). Identification of a novel 17,000-dalton parathyroid hormone-like adenylate cyclase-stimulating protein from a tumor associated with humoral hypercalcemia of malignancy. *J. Biol. Chem.*, **262**, 7151.

DEWHIRST, F.E., STASHENKO, P.P., MOLE, J.E. & TSURUMACHI, T. (1985). Purification and partial sequence of human osteoclast-activating factor: identity with interleukin I beta. *J. Immunol.*, **135**, 2562.

FELIX, R., FLEISCH, H. & ELFORD, P.R. (1989). Bone-resorbing cytokines enhance release of macrophage colony-stimulating activity by the osteoblastic cell MC3T3-E1. *Calcif. Tissue Int.*, **44**, 356.

GOLTZMAN, D., STEWART, A.F. & BROADUS, A.E. (1981). Malignancy-associated hypercalcemia: evaluation with a cytochemical bioassay for parathyroid hormone. *J. Clin. Endocrinol. Metab.*, **53**, 899.

GREAVES, M., IBBOTSON, K.J., ATKINS, D. & MARTIN, T.J. (1980). Prostaglandins as mediators of bone resorption in renal and breast tumours. *Clin. Sci.*, **58**, 201.

HABENER, J.F., ROSENBLATT, M. & POTTS, J.T. Jr (1984). Parathyroid hormone: biochemical aspects of biosynthesis, secretion, action and metabolism. *Physiol. Rev.*, **64**, 985.

HARRISON, M., JAMES, N., BROADLEY, K. & 5 others (1990). Somatostatin analogue treatment for malignant hypercalcaemia. *Br. Med. J.* (in the press).

HENDERSON, J., SHUSTIK, C., KREMER, R., RABBANI, S., HENDY, G.N. & GOLTZMAN, D. (1989). Circulating levels of parathyroid hormone-like peptide in patients with malignancy and hypercalcema. *Clin. Oncol.*, **8**, suppl. 8, abstract 30.

LORENZO, J.A., QUINTON, J., SOUSA, S. & RAISZ, L.G. (1986). Effects of DNA and prostaglandin synthesis inhibitors on the stimulation of bone resorption by epidermal growth factor in fetal rat long-bone cultures. *J. Clin. Invest.*, **77**, 1897.

MANGIN, M., IKEDA, K., DREYER, B.E. & BROADUS, A.E. (1989). isolation and characterization of the human parathyroid hormone-like peptide gene. *Proc. Natl Acad. Sci, USA*, **86**, 2408.

MANGIN, M., WEBB, A.C., DREYER, B.E. & 9 others (1988). Identification of a cDNA encoding a parathyroid hormone-like peptide from a human tumor associated with humoral hypercalcemia of malignancy. *Proc. Natl Acad. Sci. USA*, **85**, 597.

MOSELEY, J.M., KUBOTA, M., DIEFENBACH-JAGGER, H. & 8 others (1987). Parathyroid hormone-related protein purified from a human lung cancer cell line. *Proc. Natl Acad. Sci. USA*, **84**, 5048.

MUNDY, G.R., IBBOTSON, K.J., D'SOUZA, S.M., SIMPSON, E.L., JACOBS, J.W. & MARTIN, T.J. (1984). The hypercalcemia of cancer: clinical implications and pathogenic mechanisms. *N. Engl. J. Med.*, **310**, 1718.

ROSOL, T.J. & CAPEN, C.C. (1988). Pathogenesis of humoral hypercalcemia of malignancy. *Domest. Anim. Endocrinol.*, **5**, 1.

SATO, K., FUJII, Y., KASONO, K. & 6 others (1989). Parathyroid hormone-related protein and interleukin-1α synergistically stimulate bone resorption *in vitro* and increase the serum calcium concentration in mice *in vivo*. *Endocrinol.*, **124**, 2172.

SIMPSON, E.L. *et al.* (1983). Absence of parathyroid hormone messenger RNA in nonparathyroid tumors associated with hypercalcemia. *N. Engl. J. Med.*, **309**, 325.

TASHJIAN, A.H. Jr, VOELKEL, E.F., LAZZARO, M., GOAD, D., BOSMA, T. & LEVINE, L. (1987). Tumor necrosis factor-α (cachetin) stimulates bone resorption in mouse calvaria via a prostaglandin-mediated mechanism. *Endocrinology*, **120**, 2029.